# Heatwave exposure and gastrointestinal and liver diseases: complementary evidence from a population-based study and animal experiments

**DOI:** 10.3389/fphys.2026.1883010

**Published:** 2026-08-06

**Authors:** Yitian Du, Manli Wu, Haiqi Wu, Ye Wu, Cantu Fang, Luzhen Li, Huatang Zhang, Yao Wang

**Affiliations:** 1 Zhongshan Hospital of Traditional Chinese Medicine Affiliated to Guangzhou University of Traditional Chinese Medicine, Zhongshan, Guangdong, China; 2 The Tenth Clinical Medical College of Guangzhou University of Traditional Chinese Medicine, Zhongshan, Guangdong, China; 3 Shenzhen Hospital of Beijing University of Chinese Medicine (Longgang), Shenzhen, Guangdong, China; 4 School of Basic Medical Sciences, Guangzhou University of Chinese Medicine, Guangzhou, Guangdong, China; 5 State Key Laboratory of Traditional Chinese Medicine Syndrome, Guangzhou University of Chinese Medicine, Guangzhou, Guangdong, China

**Keywords:** animal experiments, CHARLS, gastrointestinal diseases, heatwave, liver diseases

## Abstract

**Background:**

Heatwaves are becoming increasingly frequent under climate change, yet their association with gastrointestinal (GI) and liver diseases remains poorly understood. This study aimed to evaluate the association between heatwave exposure and the prevalence of GI and liver diseases in middle-aged and older adults and to explore the biological plausibility using a heat-exposure mouse model.

**Methods:**

We analyzed 10,409 participants from the 2015 China Health and Retirement Longitudinal Study (CHARLS) using 12 city-level heatwave definitions and applied multivariable logistic regression models to evaluate associations between heatwave exposure and the prevalence of GI and liver diseases. A heat exposure mouse model was further used for histopathological, biochemical, immunofluorescence, inflammatory, and gut microbiome analyses.

**Results:**

Heatwave exposure was significantly associated with a higher prevalence of gastrointestinal and liver diseases in middle-aged and older adults, with a sex-divergent pattern observed in subgroup analyses. Smokers also showed stronger associations between heatwave exposure and disease prevalence. In mice, heat exposure induced gastrointestinal and hepatic injury, accompanied by dysregulated metabolism, elevated ALT, AST, TNF-α, and LPS levels, reduced colonic ZO-1 expression, and marked alterations in gut microbial composition and predicted metabolic pathways.

**Conclusion:**

These findings suggest that heatwave exposure is associated with GI and liver diseases and may be related to gut microbiome dysregulation.

## 1 Introduction

Gastrointestinal (GI) and liver diseases together constitute a major global non-communicable disease burden. These conditions not only increase mortality through acute and chronic complications but also impose a substantial and growing economic strain on healthcare systems. In the United States, GI-related healthcare expenditures reached $111.8 billion in 2021, with 281,413 associated deaths (Peery et al., 2025). Liver diseases are responsible for approximately 2 million deaths annually, accounting for 4% of global mortality (Gan et al., 2025), and their burden is projected to rise further by 2040 without effective interventions (Huang et al., 2024). In China, over 20% of the population is estimated to be affected by liver diseases, posing a significant challenge to public health (Xiao et al., 2019). Aging impacts multiple aspects of gastrointestinal function, which partly explains the higher prevalence of certain GI and liver diseases in middle-aged and older adults (Dumic et al., 2019).

Multiple factors contribute to the onset of GI and liver diseases, including irregular dietary habits, poor lifestyle patterns, and tobacco and alcohol consumption (Yuan et al., 2023). Environmental factors also play a critical role, particularly in the context of global climate change. Heatwaves—defined as periods of two or more consecutive days of unusually high temperatures—are among the most lethal forms of extreme weather. Under accelerating global warming, heatwaves have emerged as an escalating public health threat (Meehl and Tebaldi, 2004). For example, the prolonged European heatwave of 2003 caused more than 70,000 deaths (Robine et al., 2008), while the 2018 heatwave in Japan resulted in 34,147 heat-related emergency transports within an 11-day period, nearly half of whom were older adults (Hayashida et al., 2019). Over the past decade, heat-related mortality in older adults has risen by 53.7%, reaching 298,000 deaths in 2018 (Watts et al., 2021). Prolonged heat exposure is known to adversely affect cardiovascular, respiratory, and renal systems in older populations, but its relationship with GI and liver diseases remains poorly explored (Figueiredo et al., 2024). The gut–liver axis refers to the bidirectional anatomical and functional relationship between the intestine and the liver, whereby intestinal barrier integrity and gut-derived microbial products reach the liver through the portal circulation and modulate hepatic inflammation and metabolism (Tripathi et al., 2018; Hsu and Schnabl, 2023). This axis, mediated by gut microbiota metabolism and immune modulation, provides a potential mechanistic link between heat exposure and liver health (Trivedi and Adams, 2016). Previous studies have reported that heatwaves may increase the risk of acute exacerbations in patients with inflammatory bowel disease (IBD) (Manser et al., 2013). Because IBD is closely linked to liver diseases *via* the gut–liver axis, heat exposure could indirectly increase the burden of liver disease. Understanding these associations is crucial for developing effective adaptation and mitigation strategies, such as early-warning systems for heatwaves.

**Figure 1:**
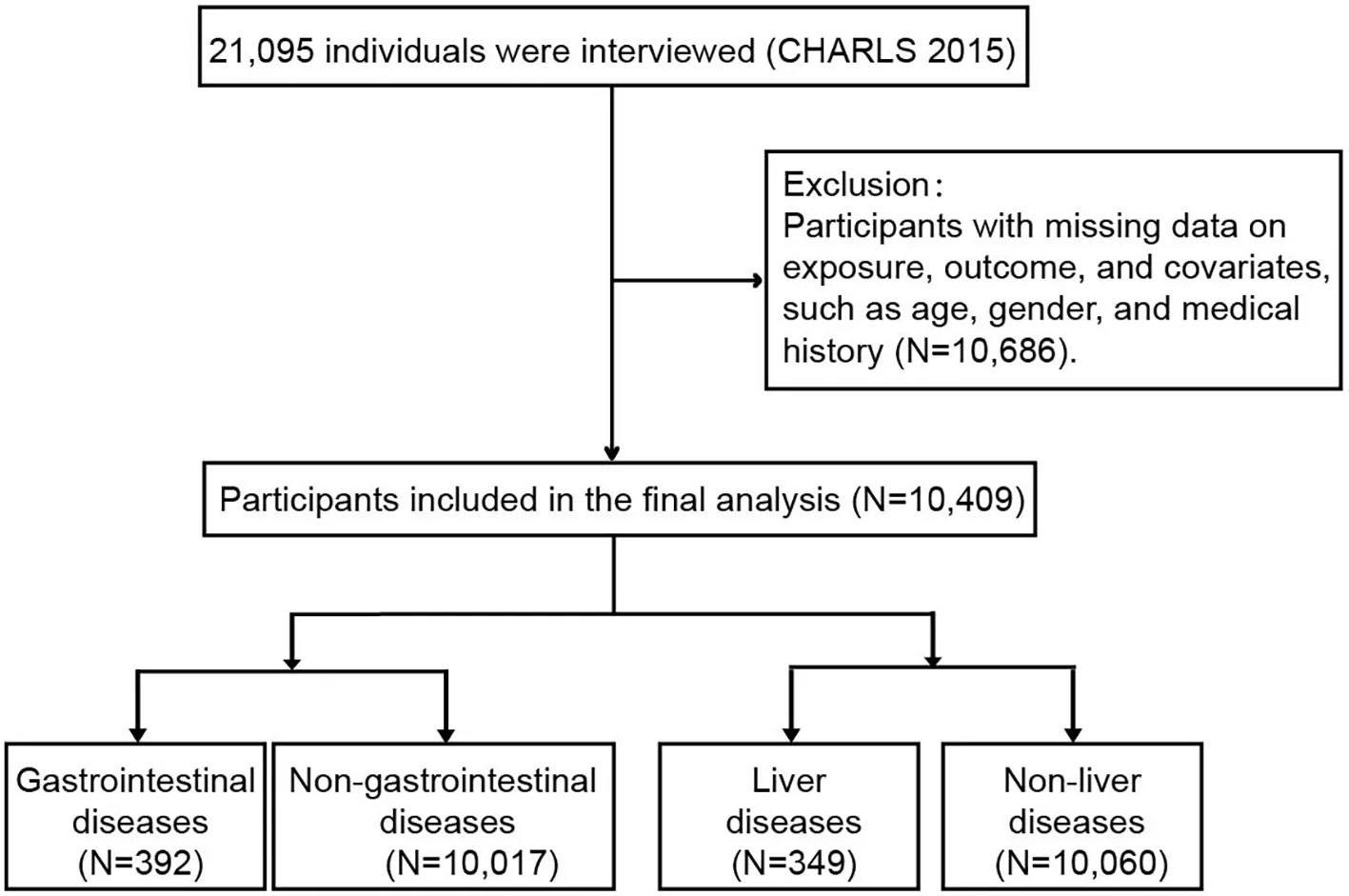
Screening flow chart of the research population.

In this study, we utilized data from the China Health and Retirement Longitudinal Study (CHARLS)—a nationally representative survey of individuals aged ≥45 years, which collects comprehensive information on socioeconomic status, health conditions, and lifestyle factors—to evaluate the association between heatwave exposure and self-reported physician-diagnosed GI and liver diseases in middle-aged and older Chinese adults. A complementary heat-exposure mouse model was used to provide biological and mechanistic evidence of heat-related gastrointestinal and hepatic injury, rather than to infer causality from the population-based analysis. The participant selection process is summarized in **Figure 1**.

## 2 Materials and methods

### 2.1 Data source and collection

This study utilized data from the CHARLS, a nationally representative, biennial survey initiated in 2011. CHARLS targets Chinese residents aged 45 years and older, covering 450 villages across 28 provinces (including autonomous regions and municipalities). The survey collects extensive information on demographics, health status, cognition, healthcare access, employment, retirement, household income, and housing conditions, providing a valuable resource for examining the health and socioeconomic conditions of middle-aged and older adults in China (Zhao et al., 2014).

### 2.2 Study design and population

Participants were selected from the 2015 CHARLS dataset. Exclusion criteria included: (1) age below 45 years, (2) missing responses to the GI and liver disease questionnaire, and (3) incomplete data on baseline characteristics or covariates. Due to the unavailability of exact residential addresses, we assessed environmental exposure at the city level, assuming uniform exposure within each city and stable residence throughout the study period. This assumption is supported by two points: first, all participants were at least 45 years old; and second, China’s household registration system restricts population mobility to some extent. The dataset used in this study was publicly available. Of the 21,095 individuals interviewed in CHARLS 2015, 10,686 with missing data on exposure, outcome, or covariates were excluded, leaving 10,409 participants in the final analytic sample. A detailed flowchart of participant selection is presented in **Figure 1**.

### 2.3 Heatwave assessment

Daily temperature data were obtained from the Data Center for Resources and Environmental Sciences, Chinese Academy of Sciences (Yang et al., 2019). For each city, data were extracted from the primary local meteorological monitoring station. Given the lack of a universally accepted heatwave definition, we used the daily minimum temperature to characterize heatwaves, as it better reflects nighttime heat stress (Xu and Tong, 2017). City-specific thresholds were defined based on the 90th, 92.5th, 95th, and 97.5th percentiles of minimum temperatures, combined with durations of ≥2, ≥3, and ≥4 consecutive days, yielding 12 heatwave definitions (**Supplementary Table 1**) (Yin et al., 2018; Xu et al., 2019). Heatwave exposure was quantified at the city level as the annual number of heatwave days and events in each participant’s city during 2015, based on the city-specific Tmin percentile and consecutive-day criteria described above. This city-level assignment may not reflect individual-level exposure due to within-city heterogeneity, mobility, indoor environment, and adaptation behaviors. Humidity and apparent temperature were not incorporated due to data limitations.

### 2.4 GI and liver diseases measurements

GI and liver disease status was determined using the Health Status and Functioning Questionnaire. Participants were asked questions such as **“**Have you been diagnosed by your doctor with a digestive disease (excluding tumors or cancers)?**”** and **“**Have you been diagnosed by your doctor with a liver disease (excluding fatty liver, cancers, and tumors)?**”** If the respondent answered **‘**yes**’**, he or she was defined as having GI or liver diseases (da007_6 and da007_10) (Chen et al., 2024). GI and liver diseases were defined based on self-reported physician diagnosis at the time of the survey. Outcomes represent ever-diagnosed status rather than newly diagnosed cases in 2015, which may compromise the temporal ordering between 2015 heatwave exposure and disease onset.

### 2.5 Covariates

Baseline covariates collected in 2015 included: age (continuous), sex (male/female), smoking status (yes/no), marital status (yes/other), education level (high/low), weight and height (continuous), body mass index (BMI)(continuous), and self-reported chronic diseases (yes/no).

### 2.6 Mice treatment

Six-week-old male C57BL/6J mice were purchased from Zhuhai BesTest Bio-Tech Co., Ltd. (Zhuhai, China; Certificate No. 44822700003283). Mice were housed in sterilized cages under standard laboratory conditions and given irradiated chow and autoclaved acidified water ad libitum. Animals were randomly assigned to either a normal control (NC) group or a heat exposure (HT) group (n = 6 per group). The NC group was maintained at 25 ± 1 °C, whereas the HT group was continuously exposed to 33 ± 1 °C for 14 consecutive days in a controlled artificial climate chamber (RXZ-158A; Ningbo Jiangnan Instrument Factory, China) (Chen et al., 2019). All mice were kept under a 12-hour light/dark cycle and monitored daily throughout the experiment. Fresh fecal samples were collected individually at the end of the exposure period for 16S rDNA sequencing. To minimize potential cage effects, mice were randomly allocated across cages within each group, and each animal was considered an independent biological replicate. After euthanasia by intraperitoneal administration of sodium pentobarbital (10 g/L, 150 mg/kg), liver, colon, pancreas, and stomach tissues were harvested for histological analysis. Serum biochemical parameters, including total cholesterol (TC), high-density lipoprotein cholesterol (HDL-C), low-density lipoprotein cholesterol (LDL-C), fasting blood glucose (FBG), alanine aminotransferase (ALT), and aspartate aminotransferase (AST), were measured using enzymatic colorimetric methods. Serum tumor necrosis factor-α (TNF-α) and lipopolysaccharide (LPS) levels were quantified using enzyme-linked immunosorbent assay (ELISA) kits (CUSABIO, Wuhan, China) according to the manufacturer’s protocols.

### 2.7 H&E Staining

Liver, colon, pancreas, and stomach tissues were fixed in 4% paraformaldehyde for 24 hours, followed by dehydration, paraffin embedding, and sectioning at a thickness of 4 μm. The sections were then subjected to routine hematoxylin and eosin (H&E) staining to evaluate histopathological changes.

### 2.8 Immunofluorescence

Colon tissues were harvested from mice, fixed in formalin, and embedded in paraffin. Tissue sections (6 μm thick) were mounted on positively charged slides, deparaffinized, and subjected to antigen retrieval in 0.01 M sodium citrate buffer (pH 6.0) for 25 min. Sections were then blocked with normal goat serum for 30 min, followed by incubation with primary antibodies at 4 °C overnight and fluorophore-conjugated secondary antibodies for 2 h at room temperature. After nuclear counterstaining with DAPI, slides were mounted with Fluoromount-G. Images were captured using a Zeiss confocal microscope.

### 2.9 16S rDNA

DNA was extracted from stool samples using the TIANamp Stool DNA Kit (Tiangen Biotech, Beijing, China). DNA collection involved bead-beating, phenol–chloroform extraction, and dissolution in Tris-EDTA (TE) buffer. DNA quality and concentration were assessed on 1% agarose gels, and samples were diluted to 1 ng/μl with sterile water. The V3–V4 regions of the bacterial 16S rRNA gene were amplified with primers 341F (CCTAYGGGRBGCASCAG) and 806R (GGACTACNNGGGTATCTAAT), incorporating barcodes (Xu et al., 2006; Ben-Othman et al., 2017). PCR was performed in 30 μL reactions containing 15 μL Phusion^®^ High-Fidelity PCR Master Mix (New England Biolabs), 0.2 μM primers, and ~10 ng DNA. Cycling conditions were: 98 °C for 1 min; 30 cycles of 98 °C for 10 s, 50 °C for 30 s, and 72 °C for 60 s; final extension at 72 °C for 5 min. PCR products were verified on 2% agarose gels, and fragments of 400–450 bp were selected. Products were pooled at equal concentrations, purified with the Qiagen Gel Extraction Kit, and libraries were prepared using the TruSeq^®^ DNA PCR-Free Kit (Illumina). Library quality was confirmed with a Qubit^®^ 2.0 Fluorometer (Thermo Scientific) and an Agilent Bioanalyzer 2100. Sequencing was conducted on the Illumina NovaSeq6000 platform, generating 250 bp paired-end reads. Microbial diversity was analyzed in R (v4.2.3). Alpha diversity indices were calculated with the vegan package (v2.6.4). Beta diversity was assessed using Principal Coordinates Analysis (PCoA) and Non-Metric Multidimensional Scaling (NMDS). Functional profiling was performed with PICRUSt2, and LEfSe analysis identified differential taxa, with an LDA score >2 considered significant.

### 2.10 Statistical analysis

Baseline characteristics were compared between participants with and without GI or liver diseases. Continuous variables were summarized as means and standard deviations (SD), and categorical variables as frequencies and percentages. Associations between heatwave exposure and disease prevalence were estimated using multivariable logistic regression models (Xue and Zhang, 2018). Exposure was modeled as the number of heatwave days (per 3-day increase) and the number of heatwave events (per one additional event). Three models were constructed: Model 1: Unadjusted. Model 2: Adjusted for age, sex, education, and marital status. Model 3: Further adjusted for smoking, weight, height, BMI, and chronic disease status. Subgroup analyses were conducted by sex and smoking status to explore effect modification. Sensitivity analyses were conducted including PM_2.5_ and O_3_ as additional covariates in Model 3 to assess the robustness of associations between heatwave exposure and gastrointestinal and liver diseases. In addition, sensitivity analyses were conducted by stratifying participants according to age groups (≥65 vs. <65 years). Separate logistic regression models were fitted within each age stratum using the same covariate adjustment strategy as in the main analysis. The 12 heatwave definitions represent correlated operationalizations of a single underlying exposure (varying only in the temperature percentile and minimum duration) rather than independent hypotheses; results are therefore reported descriptively across all definitions without formal correction for multiple comparisons, and the consistency of the direction and magnitude of associations across definitions—rather than statistical significance under any single threshold—was used to gauge robustness. Statistical significance was set at two-sided *P* < 0.05. All epidemiological analyses were performed using R software (version 4.4.1); 16S microbiome analyses were conducted in R (v4.2.3) as described in Section 2.9.

## 3 Results

### 3.1 Baseline characteristics of study participants

A total of 10,409 participants were included in the final analysis, among whom 392 had GI diseases and 349 had liver diseases. Participants were categorized at baseline according to the presence of GI and liver diseases. The mean age of the study population was 58.28 ± 10.79 years, with a higher proportion of females (76.3%). Individuals with GI or liver diseases were significantly older and reported a higher prevalence of comorbid chronic conditions than those without these diseases (*P* < 0.001). In these unadjusted univariate comparisons, GI and liver disease cases had significantly lower mean heatwave exposure (both days and events) than non-cases (**Tables 1**, **2**). In contrast, the adjusted multivariable logistic models (**Supplementary Tables 2**–**S5**) showed small but significant positive associations between per-unit increases in heatwave exposure and disease prevalence (*P* < 0.05) (**Tables 1**, **2**); this apparent discrepancy between unadjusted and adjusted estimates is addressed in the Discussion.

**Table 1:** Baseline characteristics stratified by the presence of GI diseases.

Variable	Levels	Overall	Non-GI diseases	GI diseases	*P*-value
		N =10,409	N = 10,017	N = 392	
Age (mean (SD))		58.28 (10.79)	58.20 (10.85)	60.38 (8.99)	<0.001
Gender (%)	Male	2468 (23.7)	2365 (23.6)	103 (26.3)	0.247
	Female	7941 (76.3)	7652 (76.4)	289 (73.7)	
Smoking (%)	Yes	1235 (11.9)	1196 (11.9)	39 (9.9)	0.264
	No	9174 (88.1)	8821 (88.1)	353 (90.1)	
Marriage (%)	Yes	9069 (87.1)	8731 (87.2)	338 (86.2)	0.641
	Other	1340 (12.9)	1286 (12.8)	54 (13.8)	
Weight (mean (SD))		59.21 (11.76)	59.16 (11.74)	60.35 (12.23)	0.051
Height (mean (SD))		155.55 (9.34)	155.53 (9.34)	156.02 (9.52)	0.315
Education (%)	High	8995 (86.4)	8660 (86.5)	335 (85.5)	0.625
	Low	1414 (13.6)	1357 (13.5)	57 (14.5)	
BMI (mean (SD))		24.90 (16.73)	24.89 (16.91)	25.15 (11.44)	0.763
Chronic_ diseases (%)	No	5188 (49.8)	5115 (51.1)	73 (18.6)	<0.001
	Yes	5221 (50.2)	4902 (48.9)	319 (81.4)	
Changes in the number of heatwave days (day), mean ± SD					
HW1 (90th-2D)		35.70 (41.36)	35.94 (41.42)	29.75 (39.44)	0.004
HW2 (90th-3D)		22.69 (27.20)	22.84 (27.24)	18.84 (25.87)	0.004
HW3 (90th-4D)		16.28 (20.10)	16.39 (20.13)	13.49 (19.12)	0.005
HW4 (92.5th-2D)		32.54 (39.99)	32.76 (40.05)	26.95 (38.02)	0.005
HW5 (92.5th-3D)		20.49 (26.15)	20.63 (26.20)	16.97 (24.84)	0.007
HW6 (92.5th-4D)		14.70 (19.24)	14.80 (19.27)	12.13 (18.31)	0.007
HW7 (95th-2D)		29.54 (38.59)	29.75 (38.65)	24.36 (36.67)	0.007
HW8 (95th-3D)		18.83 (25.30)	18.96 (25.34)	15.56 (24.02)	0.009
HW9 (95th-4D)		13.55 (18.55)	13.64 (18.58)	11.18 (17.63)	0.01
HW10 (97.5th-2D)		25.83 (35.89)	26.00 (35.95)	21.47 (34.09)	0.014
HW11 (97.5th-3D)		16.34 (23.21)	16.46 (23.25)	13.52 (21.93)	0.014
HW12 (97.5th-4D)		11.90 (17.19)	11.98 (17.23)	9.86 (16.17)	0.017
Changes in the number of heatwave events (number of event), mean ± SD					
HW1 (90th-2D)		4.74 (3.53)	4.76 (3.53)	4.06 (3.47)	<0.001
HW2 (90th-3D)		3.39 (2.76)	3.41 (2.76)	2.88 (2.74)	<0.001
HW3 (90th-4D)		2.52 (2.36)	2.53 (2.36)	2.11 (2.33)	0.001
HW4 (92.5th-2D)		4.40 (3.72)	4.42 (3.73)	3.69 (3.63)	<0.001
HW5 (92.5th-3D)		3.16 (2.86)	3.18 (2.86)	2.67 (2.80)	0.001
HW6 (92.5th-4D)		2.30 (2.27)	2.31 (2.27)	1.92 (2.27)	0.001
HW7 (95th-2D)		4.08 (3.77)	4.11 (3.77)	3.34 (3.70)	<0.001
HW8 (95th-3D)		2.71 (2.69)	2.73 (2.69)	2.26 (2.66)	0.001
HW9 (95th-4D)		2.14 (2.28)	2.16 (2.28)	1.75 (2.27)	<0.001
HW10 (97.5th-2D)		3.73 (3.79)	3.76 (3.79)	3.07 (3.73)	<0.001
HW11 (97.5th-3D)		2.60 (2.74)	2.62 (2.74)	2.12 (2.72)	<0.001
HW12 (97.5th-4D)		1.91 (2.25)	1.92 (2.26)	1.59 (2.18)	0.004

BMI, body mass index; GI, gastrointestinal; OR, odds ratio; 95%CI, 95% confidence interval; SD, standard deviation.

**Table 2:** Baseline characteristics stratified by liver diseases.

Variable	Levels	Overall	Non-liver diseases	Liver diseases	*P*-value
		N =10,409	N = 10,060	N = 349	
Age (mean (SD))		58.28 (10.79)	58.19 (10.84)	61.03 (8.74)	<0.001
Gender (%)	Male	2468 (23.7)	2380 (23.7)	88 (25.2)	0.543
	Female	7941 (76.3)	7680 (76.3)	261 (74.8)	
Smoking (%)	Yes	1235 (11.9)	1210 (12.0)	25 (7.2)	0.007
	No	9174 (88.1)	8850 (88.0)	324 (92.8)	
Marriage (%)	Yes	9069 (87.1)	8767 (87.1)	302 (86.5)	0.798
	Other	1340 (12.9)	1293 (12.9)	47 (13.5)	
Weight (mean (SD))		59.21 (11.76)	59.17 (11.74)	60.41 (12.35)	0.052
Height (mean (SD))		155.55 (9.34)	155.55 (9.33)	155.70 (9.71)	0.76
Education (%)	High	8995 (86.4)	8686 (86.3)	309 (88.5)	0.272
	Low	1414 (13.6)	1374 (13.7)	40 (11.5)	
BMI (mean (SD))		24.90 (16.73)	24.88 (16.87)	25.32 (12.04)	0.63
Chronic_diseases (%)	No	5188 (49.8)	5130 (51.0)	58 (16.6)	<0.001
	Yes	5221 (50.2)	4930 (49.0)	291 (83.4)	
Changes in the number of heatwave days (day), mean ± SD					
HW1 (90th-2D)		35.70 (41.36)	35.95 (41.47)	28.43 (37.46)	0.001
HW2 (90th-3D)		22.69 (27.20)	22.86 (27.27)	17.94 (24.52)	0.001
HW3 (90th-4D)		16.28 (20.10)	16.40 (20.15)	12.83 (18.11)	0.001
HW4 (92.5th-2D)		32.54 (39.99)	32.78 (40.10)	25.62 (35.99)	0.001
HW5 (92.5th-3D)		20.49 (26.15)	20.65 (26.23)	16.09 (23.47)	0.001
HW6 (92.5th-4D)		14.70 (19.24)	14.81 (19.29)	11.47 (17.30)	0.001
HW7 (95th-2D)		29.54 (38.59)	29.77 (38.70)	23.00 (34.61)	0.001
HW8 (95th-3D)		18.83 (25.30)	18.98 (25.37)	14.64 (22.65)	0.002
HW9 (95th-4D)		13.55 (18.55)	13.65 (18.61)	10.49 (16.60)	0.002
HW10 (97.5th-2D)		25.83 (35.89)	26.03 (36.00)	20.11 (32.05)	0.002
HW11 (97.5th-3D)		16.34 (23.21)	16.47 (23.28)	12.63 (20.57)	0.002
HW12 (97.5th-4D)		11.90 (17.19)	11.99 (17.25)	9.20 (15.14)	0.003
Changes in the number of heatwave events (number of event), mean ± SD					
HW1 (90th-2D)		4.74 (3.53)	4.76 (3.53)	4.03 (3.49)	<0.001
HW2 (90th-3D)		3.39 (2.76)	3.41 (2.76)	2.84 (2.73)	<0.001
HW3 (90th-4D)		2.52 (2.36)	2.53 (2.36)	2.07 (2.29)	<0.001
HW4 (92.5th-2D)		4.40 (3.72)	4.42 (3.73)	3.66 (3.61)	<0.001
HW5 (92.5th-3D)		3.16 (2.86)	3.17 (2.86)	2.64 (2.77)	0.001
HW6 (92.5th-4D)		2.30 (2.27)	2.31 (2.27)	1.89 (2.24)	0.001
HW7 (95th-2D)		4.08 (3.77)	4.10 (3.77)	3.30 (3.66)	<0.001
HW8 (95th-3D)		2.71 (2.69)	2.73 (2.70)	2.22 (2.61)	<0.001
HW9 (95th-4D)		2.14 (2.28)	2.16 (2.28)	1.71 (2.22)	<0.001
HW10 (97.5th-2D)		3.73 (3.79)	3.76 (3.79)	3.03 (3.69)	<0.001
HW11 (97.5th-3D)		2.60 (2.74)	2.62 (2.74)	2.07 (2.67)	<0.001
HW12 (97.5th-4D)		1.91 (2.25)	1.92 (2.26)	1.54 (2.12)	0.002

BMI, body mass index; GI, gastrointestinal; OR, odds ratio; 95%CI, 95% confidence interval; SD, standard deviation.

### 3.2 Effects of heatwave exposure on GI and liver diseases

**Figure 2:**
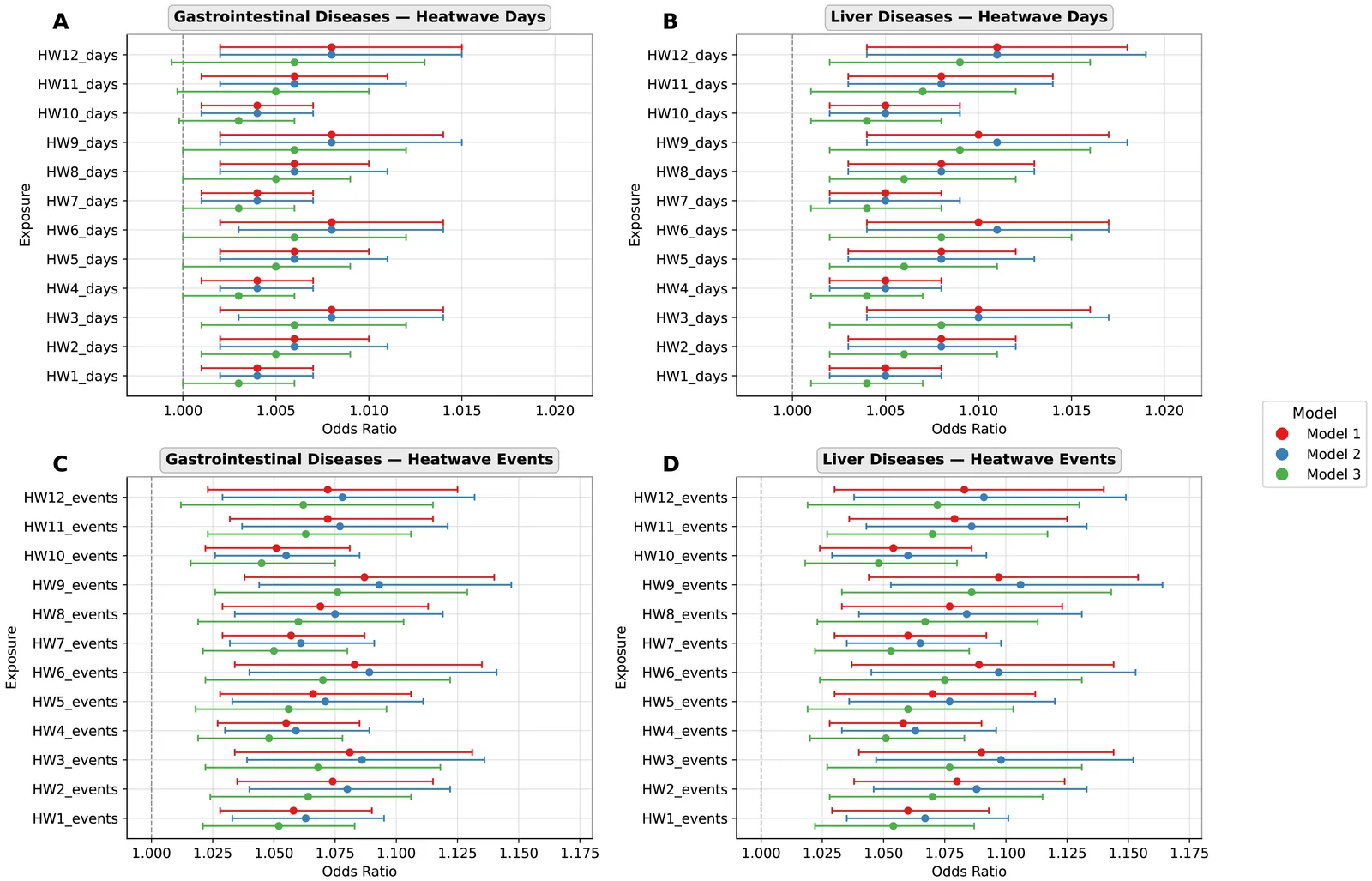
Adjusted OR and 95% CI of GI and liver diseases in relation to heatwave days and events. **(A)** Heatwave days and GI diseases. **(B)** Heatwave days and liver diseases. **(C)** Heatwave events and GI diseases. **(D)** Heatwave events and liver diseases. Note: Model 1 is unadjusted; Model 2 is adjusted for gender, age, marital status, and education; Model 3 is further adjusted for smoking, weight, height, BMI, and self-reported chronic diseases. GI, gastrointestinal; OR: odds ratio; CI, confidence intervals.

**Figure 2** and **Supplementary Tables 2**-**S5** present the associations between heatwave days/events and the prevalence of GI and liver diseases. In the fully adjusted Model 3, no significant association was observed between GI disease prevalence and the number of heatwave days under definitions HW10, HW11, and HW12 (**Figure 2A**, **Supplementary Table 2**). However, positive associations were consistently identified for heatwave days defined by HW1–HW9 and for all heatwave definitions in relation to liver disease prevalence (P < 0.05), with odds ratios (ORs) greater than 1 across all three models. The ORs and 95% confidence intervals (CIs) in Model 3 remained largely unchanged after additional adjustment for PM_2.5_ and O_3_ (**Supplementary Figure 1**), indicating the robustness of these associations. Overall, these findings suggest that heatwave exposure is associated with a higher prevalence of GI and liver diseases.

### 3.3 Subgroup analysis

**Figure 3:**
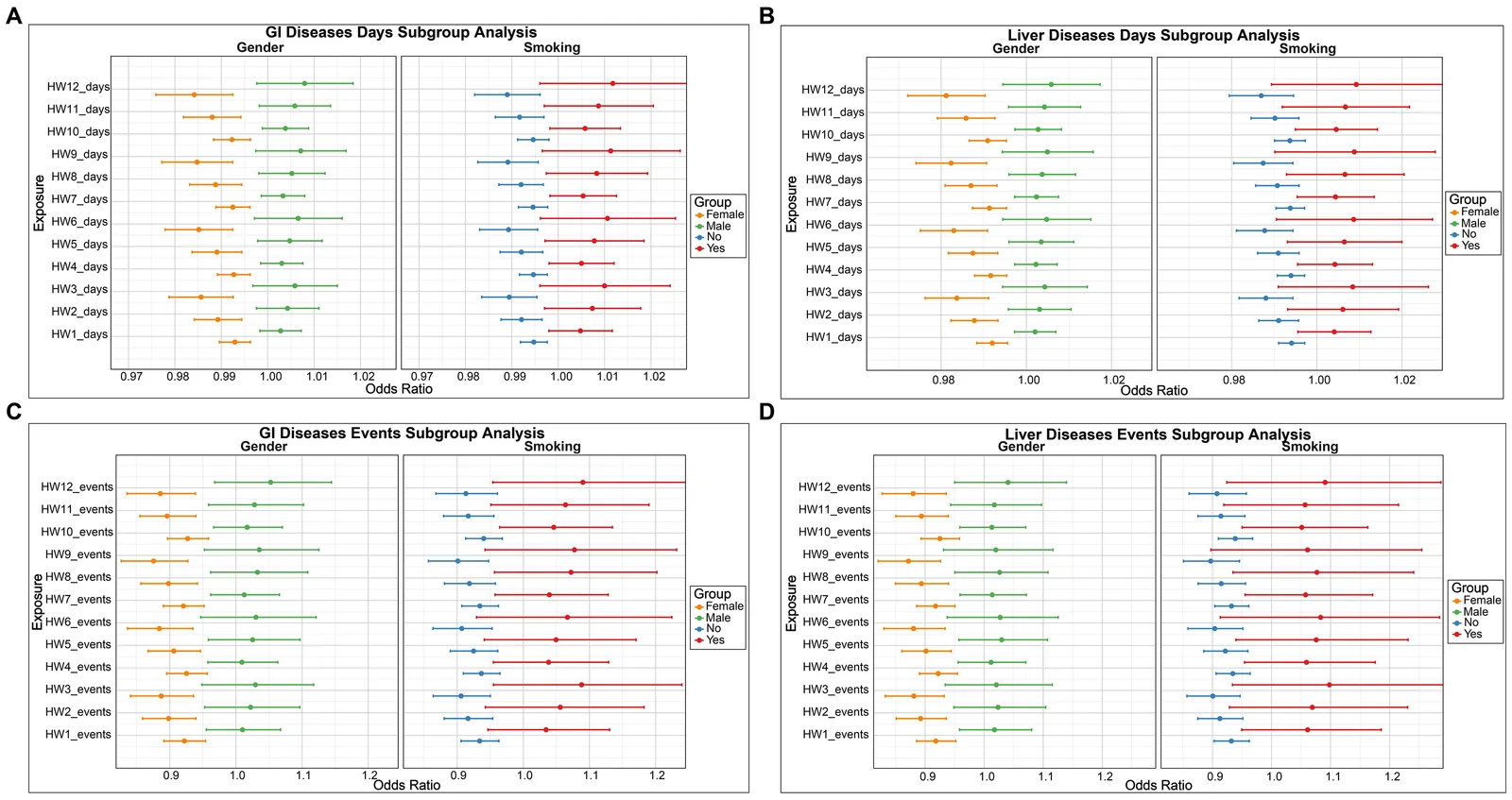
Association between heatwave exposure and GI and liver diseases, stratified by participant gender and smoking status. **(A)** Heatwave days and GI diseases. **(B)** Heatwave days and liver diseases. **(C)** Heatwave events and GI diseases. **(D)** Heatwave events and liver diseases. GI, gastrointestinal.

To further explore the association between heatwave exposure and the prevalence of GI and liver diseases, subgroup analyses were performed according to sex and smoking status. These analyses revealed a sex-divergent pattern (**Figure 3**): in men, heatwave exposure—particularly the number of heatwave events—was associated with a higher prevalence of GI and liver diseases (OR > 1), whereas in women the associations were inverse (OR < 1). In addition, smokers showed stronger associations between heatwave exposure and the prevalence of GI and liver diseases than non-smokers, with positive associations observed for both heatwave days and heatwave events. The potential biological and behavioral mechanisms underlying these sex-specific differences are discussed in the Discussion section (**Figure 3**).

### 3.4 Sensitivity analyses

To evaluate the robustness of the main findings, we performed age-stratified sensitivity analyses in which GI or liver disease was modeled as the outcome using Model 3-adjusted logistic regression conducted separately within the <65-year and ≥65-year strata (with the same covariates as Model 3, excluding age, which defined the strata). Across all twelve heatwave definitions, the per-unit associations within each stratum were modest and close to the null. Inverse associations (ORs slightly below 1) reached statistical significance mainly in the <65-year stratum. For example, for GI disease, the OR was 0.994 (95% CI: 0.989–0.999) per additional HW2 day and 0.932 (95% CI: 0.889–0.977) per additional HW2 event, whereas the corresponding associations in the ≥65-year stratum were of similar direction but did not reach statistical significance (e.g., OR = 0.997, 95% CI: 0.990–1.004 per additional HW2 day). A comparable pattern was observed for liver disease (**Table 3**). These stratum-specific estimates differed slightly in direction from the small positive associations observed in the full-sample adjusted models (**Supplementary Tables 2**–**S5**), which may reflect the strong age structure of the cohort and the very small effect sizes involved (approximately 1% per heatwave day and 5–9% per heatwave event). Taken together, the sensitivity analyses suggest that the overall heatwave–disease association is modest in magnitude and that the direction of the per-unit estimates is sensitive to age stratification, as discussed in the Discussion section.

**Table 3:** Age-stratified associations between heatwave exposure and GI and liver diseases (Model 3–adjusted logistic regression).

Exposure	Age <65 OR (95% CI)	*P*	Age ≥65 OR (95% CI)	*P*
HW1 days	0.996 (0.993–1.000)	0.025	0.998 (0.993–1.003)	0.372
HW2 days	0.994 (0.989–0.999)	0.029	0.997 (0.990–1.004)	0.392
HW3 days	0.992 (0.985–0.999)	0.034	0.996 (0.986–1.005)	0.388
HW4 days	0.996 (0.993–1.000)	0.031	0.998 (0.993–1.003)	0.399
HW5 days	0.994 (0.989–1.000)	0.039	0.997 (0.990–1.004)	0.424
HW6 days	0.992 (0.985–1.000)	0.042	0.996 (0.986–1.006)	0.418
HW7 days	0.996 (0.992–1.000)	0.040	0.998 (0.993–1.003)	0.420
HW8 days	0.994 (0.989–1.000)	0.049	0.997 (0.990–1.005)	0.459
HW9 days	0.992 (0.985–1.000)	0.053	0.996 (0.986–1.007)	0.463
HW10 days	0.996 (0.992–1.000)	0.064	0.998 (0.993–1.004)	0.558
HW11 days	0.994 (0.988–1.000)	0.065	0.998 (0.989–1.006)	0.562
HW12 days	0.992 (0.984–1.001)	0.072	0.997 (0.986–1.008)	0.598

Exposure	Age <65 OR (95% CI)	*P*	Age ≥65 OR (95% CI)	*P*
HW1 events	0.945 (0.912–0.980)	0.002	0.955 (0.908–1.005)	0.075
HW2 events	0.932 (0.889–0.977)	0.004	0.947 (0.887–1.011)	0.103
HW3 events	0.930 (0.879–0.983)	0.010	0.942 (0.871–1.018)	0.130
HW4 events	0.949 (0.916–0.982)	0.003	0.958 (0.913–1.005)	0.082
HW5 events	0.938 (0.895–0.982)	0.006	0.957 (0.899–1.020)	0.175
HW6 events	0.927 (0.874–0.982)	0.011	0.942 (0.870–1.020)	0.141
HW7 events	0.947 (0.914–0.981)	0.002	0.956 (0.912–1.003)	0.067
HW8 events	0.935 (0.890–0.983)	0.008	0.953 (0.891–1.019)	0.161
HW9 events	0.921 (0.868–0.978)	0.007	0.939 (0.866–1.018)	0.125
HW10 events	0.949 (0.916–0.983)	0.004	0.966 (0.921–1.013)	0.152
HW11 events	0.932 (0.888–0.979)	0.005	0.949 (0.888–1.015)	0.125
HW12 events	0.933 (0.879–0.992)	0.025	0.950 (0.875–1.031)	0.217

Exposure	Age <65 OR (95% CI)	*P*	Age ≥65 OR (95% CI)	*P*
HW1 days	0.995 (0.992–0.999)	0.013	0.997 (0.992–1.002)	0.207
HW2 days	0.993 (0.987–0.999)	0.014	0.995 (0.988–1.003)	0.215
HW3 days	0.991 (0.983–0.998)	0.017	0.994 (0.984–1.004)	0.216
HW4 days	0.995 (0.991–0.999)	0.015	0.997 (0.992–1.002)	0.222
HW5 days	0.993 (0.987–0.999)	0.018	0.995 (0.988–1.003)	0.238
HW6 days	0.990 (0.982–0.998)	0.019	0.994 (0.983–1.004)	0.234
HW7 days	0.995 (0.991–0.999)	0.017	0.997 (0.991–1.002)	0.233
HW8 days	0.993 (0.986–0.999)	0.020	0.995 (0.987–1.003)	0.257
HW9 days	0.990 (0.981–0.998)	0.021	0.994 (0.983–1.005)	0.256
HW10 days	0.995 (0.990–0.999)	0.024	0.997 (0.991–1.003)	0.322
HW11 days	0.992 (0.985–0.999)	0.024	0.996 (0.987–1.004)	0.320
HW12 days	0.989 (0.980–0.999)	0.027	0.994 (0.982–1.006)	0.342

Exposure	Age <65 OR (95% CI)	*P*	Age ≥65 OR (95% CI)	*P*
HW1 events	0.945 (0.909–0.982)	0.004	0.953 (0.904–1.005)	0.073
HW2 events	0.928 (0.882–0.976)	0.004	0.942 (0.880–1.009)	0.088
HW3 events	0.922 (0.868–0.979)	0.008	0.937 (0.864–1.016)	0.113
HW4 events	0.948 (0.913–0.984)	0.005	0.955 (0.908–1.004)	0.073
HW5 events	0.936 (0.891–0.983)	0.009	0.954 (0.893–1.019)	0.159
HW6 events	0.924 (0.867–0.983)	0.013	0.937 (0.863–1.018)	0.123
HW7 events	0.945 (0.910–0.982)	0.004	0.955 (0.908–1.003)	0.065
HW8 events	0.929 (0.880–0.980)	0.007	0.950 (0.885–1.018)	0.147
HW9 events	0.912 (0.854–0.972)	0.005	0.934 (0.859–1.016)	0.110
HW10 events	0.947 (0.911–0.983)	0.005	0.964 (0.918–1.012)	0.142
HW11 events	0.926 (0.879–0.977)	0.005	0.945 (0.882–1.013)	0.110
HW12 events	0.923 (0.864–0.986)	0.017	0.945 (0.868–1.029)	0.194

OR, odds ratio; CI, confidence interval. Models adjusted as in Model 3 (age, sex, education, marital status, smoking, weight, height, BMI, chronic-disease status), stratified by age (<65 vs ≥65 years). HW1–HW12 denote the twelve heatwave definitions (see **Supplementary Table 1**).

### 3.5 Heat exposure induces metabolic abnormalities and exacerbates pathological alterations in the gastrointestinal tract and liver

**Figure 4:**
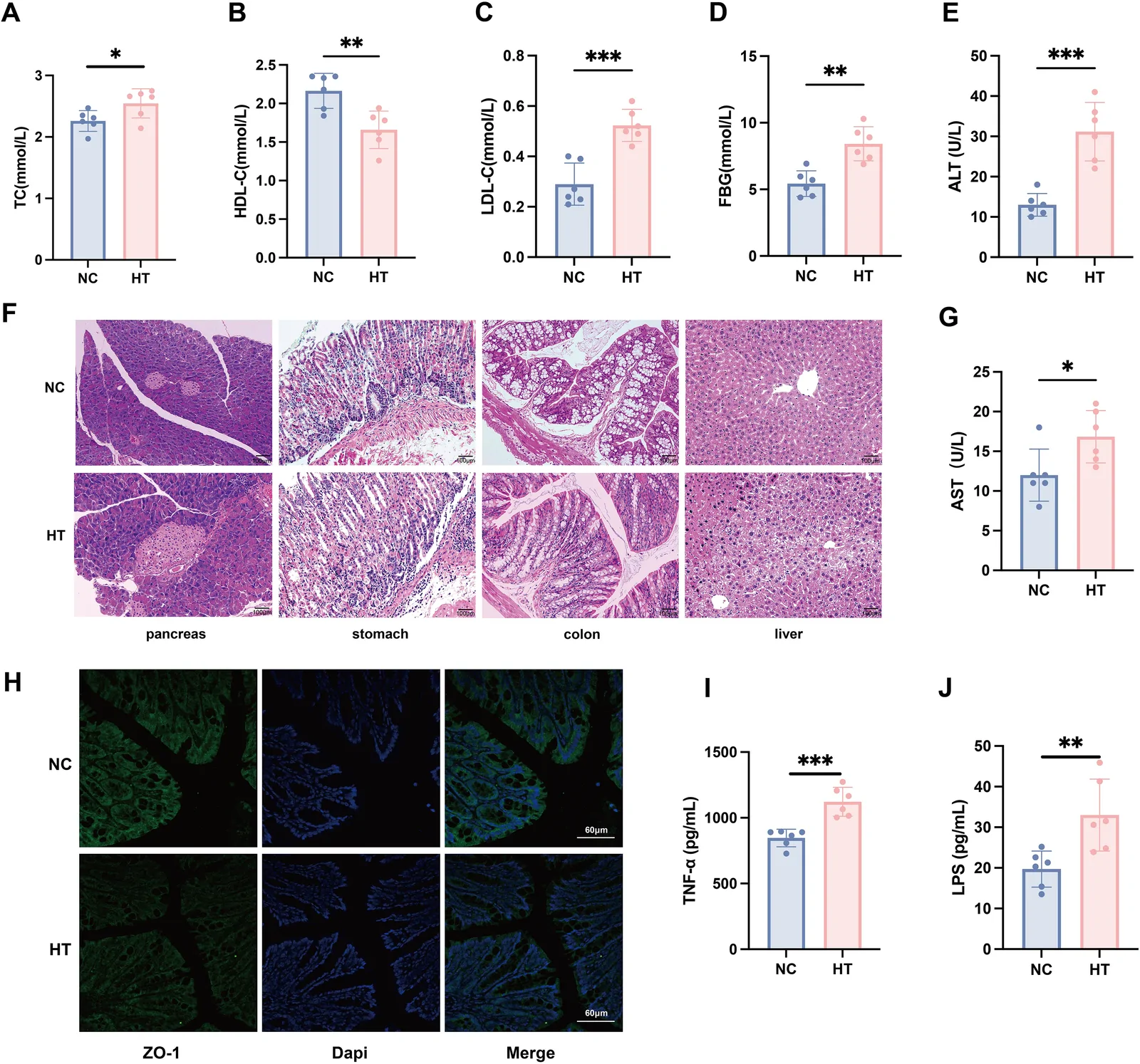
Heat exposure induces metabolic abnormalities, tissue injury, intestinal barrier impairment, and systemic inflammation in mice. **(A–D)** Serum levels of TC, HDL-C, LDL-C, and FBG in normal control (NC) and heat-treated (HT) mice. **(E, G)** Serum ALT and AST levels. **(F)** Representative H&E staining images of pancreas, stomach, colon, and liver tissues from NC and HT mice. Scale bars = 100 μm. **(H)** IF staining of ZO-1 in colon tissues. Green indicates ZO-1 and blue indicates DAPI. Scale bars = 60 μm. **(I, J)** Serum TNF-α and LPS levels in NC and HT mice. Data are presented as mean ± SD (n = 6 per group). Statistical significance was determined using Student’s t-test. ^*^P < 0.05, ^**^P < 0.01, ^***^P < 0.001. TC, total cholesterol; HDL-C, high-density lipoprotein cholesterol; LDL-C, low-density lipoprotein cholesterol; FBG, fasting blood glucose; ALT, alanine aminotransferase; AST, aspartate aminotransferase; TNF-α, tumor necrosis factor-α; LPS, lipopolysaccharide; IF, immunofluorescence.

To examine the biological plausibility of the heat–disease associations observed in the human cohort under controlled conditions, we further assessed metabolic parameters, tissue pathology, intestinal barrier integrity, and inflammatory cytokines in mice. Compared with the NC group, HT mice showed significant alterations in metabolic profiles, including lower HDL-C levels and higher TC, LDL-C, and FBG levels (**Figures 4A–D**), suggesting disrupted lipid and glucose metabolism. Heat exposure also induced evident liver injury, as reflected by significantly increased serum ALT and AST levels (**Figures 4E, G**). Compared to the NC group, the HT group exhibited evident hepatocellular swelling, mild steatosis, and nuclear pyknosis (**Figure 4F**). In the colon, the HT group showed pronounced crypt atrophy, a marked reduction in goblet cell numbers, and substantial infiltration of inflammatory cells (**Figure 4F**). Pancreatic tissues in the NC group displayed a compact and organized structure, whereas the HT group showed disrupted architecture and cellular swelling (**Figure 4F**). Moreover, extensive inflammatory infiltration was observed in the gastric tissue of HT mice (**Figure 4F**).

We further assessed colonic tight junction integrity. Immunofluorescence staining revealed a marked reduction in zonula occludens-1 (ZO-1) expression in HT mice compared with NC mice (**Figure 4H**), indicating impairment of the intestinal epithelial barrier. Moreover, serum TNF-α and LPS levels were significantly elevated in the HT group (**Figures 4I, J**), suggesting that heat exposure promoted systemic inflammation and endotoxemia. Collectively, these findings indicate that heat exposure induces intestinal barrier dysfunction and inflammatory injury accompanied by hepatic damage. These barrier and inflammatory changes are consistent with, but do not by themselves establish, a gut–liver axis contribution to the heatwave–disease association observed in the cohort.

### 3.6 Heat exposure alters the gut microbiome in mice

**Figure 5:**
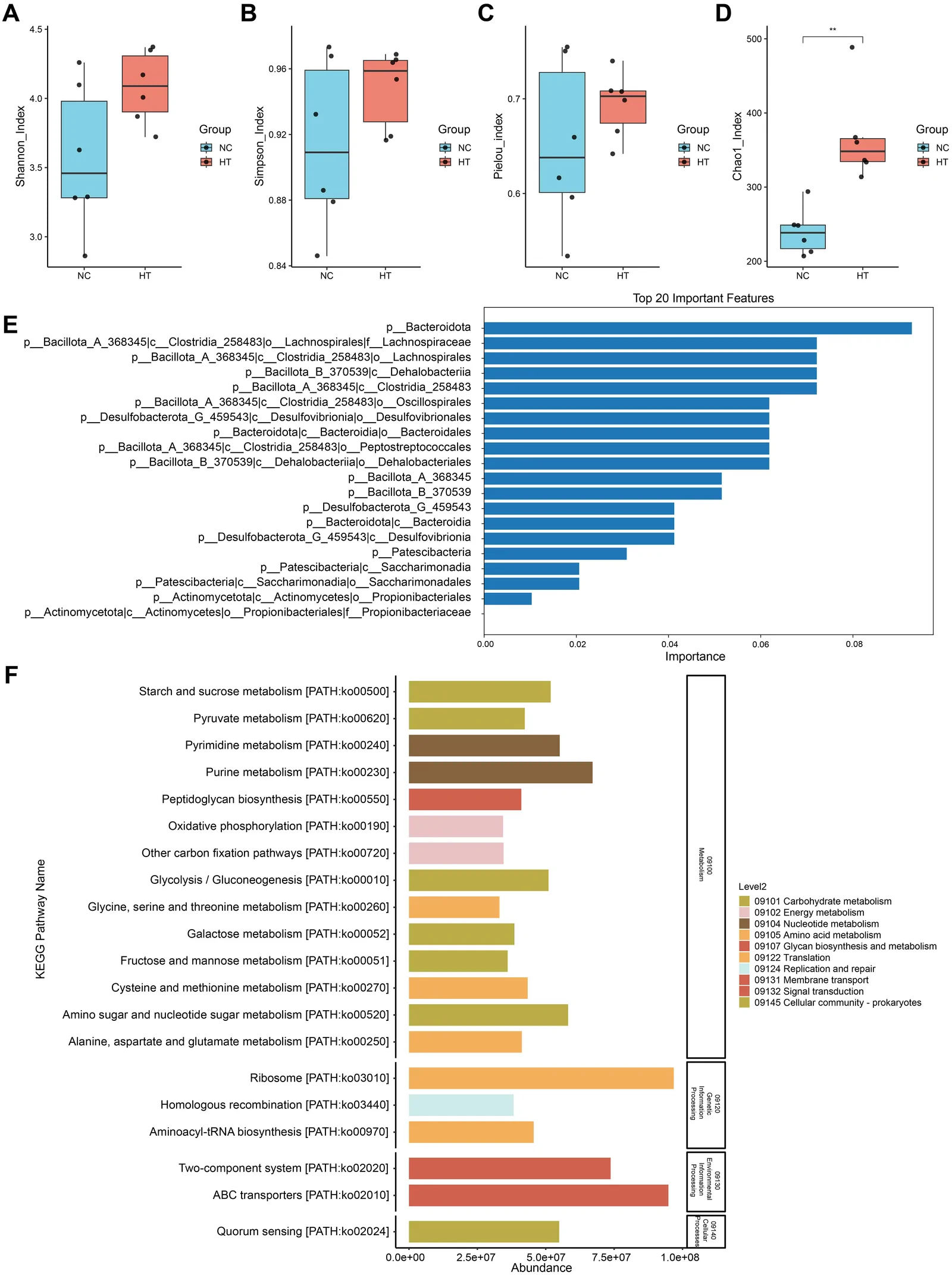
Results of 16S rDNA sequencing. **(A)** Shannon index. **(B)** Simpson index. **(C)** Pielou index. **(D)** Chao1 index. ^*^P < 0.05. **(E)** Random Forest analysis. The x-axis represents the importance scores of taxa in the classifier model, and the y-axis indicates taxonomic units at the phylum. From top to bottom, taxa are ranked in descending order of their contribution to group discrimination; those with higher importance can be considered potential biomarkers distinguishing the groups. **(F)** KEGG functional analysis. The x-axis denotes the relative abundance of functional pathways, and the y-axis shows the KEGG pathways. The rightmost column indicates the corresponding first-level pathway, while colors represent different second-level pathways. The figure presents the top 20 pathways ranked by total abundance across all samples. KEGG, Kyoto Encyclopedia of Genes and Genomes.

To explore the potential mechanisms underlying heat-induced gastrointestinal and hepatic injury, we examined gut microbiome alterations using 16S rDNA sequencing. Alpha diversity analysis showed that the Shannon (**Figure 5A**), Simpson (**Figure 5B**), Pielou (**Figure 5C**), and Chao1 (**Figure 5D**) indices were higher in the HT group than in the NC group, with the increase in the Chao1 index reaching statistical significance. Taxonomic analysis revealed that Bacteroidota, Bacteroidia, Bacteroidales, Patescibacteria, Saccharimonadia, and Saccharimonadales were enriched after HT treatment (**Supplementary Figure 2**). Furthermore, Random Forest analysis (Breiman, 2001) demonstrated that gut microbiome features could accurately distinguish NC from HT mice, indicating marked heat-induced alterations in gut microbiome composition (**Figure 5E**). To further characterize the functional potential of the microbiome, Kyoto Encyclopedia of Genes and Genomes (KEGG) functional profiling identified 20 highly abundant pathways, including alanine, aspartate and glutamate metabolism, amino sugar and nucleotide sugar metabolism, fructose and mannose metabolism, galactose metabolism, and oxidative phosphorylation (**Figure 5F**). Because **Figure 5F** represents total pathway abundance rather than a formal between-group enrichment analysis, these pathways are reported as descriptive features rather than statistically enriched or mediating factors.

## 4 Discussion

Our study provides timely real-world evidence supporting the association between heatwave exposure and the prevalence of GI and liver diseases in middle-aged and older adults. First, we observed that heatwave events were associated with a higher prevalence of both GI and liver diseases. Second, multivariable logistic regression revealed positive associations between the number of heatwave days and liver disease prevalence. However, no significant association was observed between GI disease prevalence and high-intensity heatwave days (HW10–HW12), after adjusting for potential confounders such as smoking status, body weight, height, BMI, and self-reported chronic conditions. Furthermore, subgroup analyses indicated that sex and smoking status significantly modified the associations between heatwave exposure and the prevalence of GI and liver diseases.

An apparent discrepancy between the unadjusted and adjusted estimates warrants comment. In the crude baseline comparison, disease cases had lower mean heatwave exposure than non-cases (**Tables 1**, **2**), whereas the multivariable models revealed small positive associations between heatwave exposure and disease prevalence (**Supplementary Tables 2**–**S5**). This reversal is characteristic of negative confounding: disease cases were substantially older and far more likely to report comorbid chronic conditions, factors that are correlated with both the measured heatwave exposure and disease prevalence. After adjustment for age, chronic disease status, and the remaining covariates, the underlying positive association between heatwave exposure and disease prevalence emerged. Consistent with the modest magnitude of this association, the age-stratified sensitivity analyses (**Table 3**) produced per-unit estimates close to the null within strata. We therefore interpret the heatwave–disease association as real but small in magnitude, and we caution against over-interpreting the direction of individual per-unit estimates, which is sensitive to model specification and to the strong age structure of the cohort.

Climate change has led to a rise in extreme weather events, which are anticipated to exert an increasingly significant impact on human health. Previous studies have provided preliminary evidence of the association between high-temperature and humidity exposure and adverse health outcomes (Hashiguchi et al., 2013; Goad and Gawkrodger, 2016; Sobolewski et al., 2021; Fang et al., 2023; Mei et al., 2023). Under high-temperature conditions, evaporative cooling is the primary thermoregulatory mechanism in humans, and its efficiency depends on the humidity gradient between the skin and the surrounding atmosphere. When heat dissipation is impaired, core body temperature rises, potentially resulting in severe health consequences. Acute heat-related illnesses such as heat exhaustion and heatstroke can develop rapidly—within hours to days—and are characterized by symptoms including excessive sweating, nausea, vomiting, and syncope. In addition, heat exposure can indirectly aggravate pre-existing conditions, such as cardiovascular and kidney diseases, which often exhibit delayed health effects over several days (Mora et al., 2017). Symptoms such as nausea, vomiting, and syncope are common in both heat-related illnesses (e.g., heat exhaustion and heatstroke) and GI and liver diseases (Epstein and Yanovich, 2019; Zhang et al., 2024), and in GI and liver diseases. However, limited evidence exists regarding the association between high temperature exposure and GI and liver diseases, particularly in terms of long-term effects. This nationally representative design provides comprehensive evidence on the potential health burden of heatwave exposure in middle-aged and older adults. In this analysis, heatwave exposure was examined as a potential environmental factor associated with the prevalence of GI and liver diseases, with particular attention to vulnerable subgroups. Notably, individuals with a history of smoking showed stronger associations between heatwave exposure and prevalent GI and liver diseases under high-temperature conditions. Furthermore, we explored the modifying effects of sex and smoking status on the association between heatwave exposure and the prevalence of GI and liver diseases. Subgroup analyses revealed a sex-divergent association between heatwave exposure and the prevalence of GI and liver diseases. Several non-exclusive mechanisms may contribute to this pattern: men in this cohort were more likely to smoke and may have greater outdoor or occupational heat exposure, whereas the inverse associations observed in women could reflect differences in healthcare-seeking behavior, indoor time, or residual confounding. Prior research has nonetheless reported that women can be more vulnerable to certain heat-related outcomes; for example, heatwaves have been linked to increased hospital admissions for pneumonia, particularly among females (Wu et al., 2024). In addition, individuals with a history of smoking appeared more susceptible to heatwave-associated GI and liver diseases than non-smokers. This heightened susceptibility may be related to the detrimental impact of smoking on gastrointestinal epithelial regeneration (Wu and Cho, 2004; Zhang et al., 2012). Smoking has also been associated with an increased risk of *Helicobacter pylori* infection (Endoh and Leung, 1994; Arkkila et al., 2007).

Mounting evidence suggests that smoking is associated with the progression of chronic liver diseases through multiple mechanisms (Marti-Aguado et al., 2022). Smoking has been associated with hepatic steatosis and may be linked to more severe liver injury in the context of non-alcoholic steatohepatitis (NASH) (Savari et al., 2019). Furthermore, several toxic compounds in tobacco smoke—such as 4-aminobiphenyl and polycyclic aromatic hydrocarbons—are metabolized in the liver into reactive carcinogenic intermediates (Chen et al., 2002; Sugamori et al., 2012). These substances have been reported to induce histopathological alterations, including hepatocellular degeneration, in experimental animal models (Balansky et al., 2012). The Intergovernmental Panel on Climate Change (IPCC) Assessment Report 2021 forecasts a rising frequency of extreme environmental events globally, including droughts, heatwaves, and bushfires (Intergovernmental Panel on Climate Change, 2022). Elevated temperatures have been shown to negatively impact human health, particularly by increasing the risk of cardiovascular mortality (Wu et al., 2025). Consistent with recent global assessments of the heat-related health burden (Al Ali et al., 2026), our findings extend the spectrum of heat-sensitive conditions to include gastrointestinal and hepatic outcomes. From a translational standpoint, they reinforce the importance of heatwave preparedness, vulnerability assessment, and adaptation planning for susceptible populations, including heat–health early-warning systems (Dube et al., 2025). As a coping strategy, the use of air conditioning has become increasingly prevalent in response to high temperatures. In China, indoor smoking commonly occurs in enclosed spaces equipped with split-type air conditioners—such as living rooms, studies, and bedrooms—where smokers often keep doors and windows closed while the air conditioning is operating. This behavior may deteriorate indoor air quality by elevating PM2.5 concentrations (Xie et al., 2022). Substantial evidence has demonstrated that PM2.5 exposure poses considerable health risks and is associated with various chronic conditions (Chen et al., 2024; Lin et al., 2025). These factors may contribute to the stronger associations observed between smoking and prevalent GI and liver diseases during heatwave conditions. We note that, in the unadjusted baseline data, the proportion of smokers was lower among disease cases (**Table 2**), which may reflect reverse causation, such as smoking cessation after diagnosis. Therefore, the smoking-related associations are better supported by the subgroup analyses than by the baseline comparisons. Moreover, because most smokers in this cohort were male, the combined influence of heatwave exposure and smoking may partially account for the observed sex-specific differences. Taken together, the co-existence of heatwave exposure and smoking may exert synergistic adverse effects on human health.

Accumulating evidence indicates that gut microbiota plays a pivotal role in the pathogenesis of gastrointestinal and liver diseases through complex host–microbe interactions. Dysbiosis, characterized by reduced microbial diversity and functional imbalance, has been consistently observed in IBD, colorectal cancer (CRC), nonalcoholic fatty liver disease (NAFLD), and cirrhosis (Wong and Yu, 2023; Al-Busafi et al., 2025). For example, *Fusobacterium nucleatum* has been implicated in CRC development, whereas the loss of butyrate-producing bacteria such as *Faecalibacterium prausnitzii* is linked to IBD (Kostic et al., 2013). In liver diseases, microbial translocation across a compromised intestinal barrier promotes endotoxemia and hepatic inflammation *via* the gut–liver axis (Albillos et al., 2020). Moreover, recent studies suggest that specific microbiota signatures may serve as non-invasive biomarkers for disease diagnosis and prognosis, while interventions such as diet, probiotics, and fecal microbiota transplantation hold therapeutic potential, although clinical translation remains limited (Allegretti et al., 2019; Schirmer et al., 2019). Collectively, these findings highlight the gut microbiome as both a driver of GI and liver pathology and a promising target for precision medicine. We found that heatwave exposure increased the abundance of *Bacteroidota*, *Bacteroidales*, *Bacteroidia*, *Patescibacteria*, *Saccharimonadia* and *Saccharimonadales*. Importantly, several of these microbial taxa have been implicated in GI and liver diseases. Members of the *Bacteroidota*, particularly those within the *Bacteroidia* and the *Bacteroidales*, are frequently altered in colorectal cancer and inflammatory bowel disease, where they have been associated with epithelial barrier disruption, pro-inflammatory responses, and tumor-promoting activities (Kostic et al., 2012; Wong and Yu, 2019; Xu et al., 2025). Similarly, *Bacteroidota* have been linked to hepatic disorders such as non-alcoholic fatty liver disease (NAFLD) and cirrhosis, potentially through modulation of bile acid metabolism and endotoxin production (Qin et al., 2014; Boursier et al., 2016). In contrast, *Patescibacteria* and its representatives *Saccharimonadia* and *Saccharimonadales*, although less studied, have recently been reported to show increased abundance in colorectal cancer and liver fibrosis, suggesting that these underexplored taxa may also contribute to host metabolic dysregulation and immune perturbation (Brown et al., 2015; Wong and Yu, 2019; Iwaki et al., 2021). Together, these observations suggest that the heatwave-associated shifts in these microbial groups may be associated with susceptibility to GI and liver pathologies.

This study has several limitations that should be considered. First, the molecular mechanisms linking high-temperature exposure to the higher prevalence of GI and liver diseases were not explored. Second, due to the lack of questionnaire data on conditions such as fatty liver, tumors, and cancer, these diseases were not included in our analysis, although they merit further investigation. Third, information on pre-existing gastrointestinal and liver conditions was not incorporated into the baseline longitudinal analysis, which should be addressed in future studies. Additionally, the GI and liver disease outcomes were based on self-reported physician diagnosis, reflecting prevalent or ever-diagnosed conditions rather than incident cases in 2015. As a result, the temporal relationship between 2015 heatwave exposure and disease onset cannot be firmly established, and the observed associations should be interpreted as correlations rather than causal effects. Exposure assignment at the city level may not capture individual variation in heat stress, and daily minimum temperature alone may not fully reflect physiologically relevant heat exposure. Relatedly, the lower mean heatwave exposure among disease cases in the unadjusted comparisons (**Tables 1**, **2**), alongside the small positive associations in the adjusted models (**Supplementary Tables 2**–**S5**), likely reflects geographic confounding, as heatwave-prone regions differ systematically in demographic and healthcare-access characteristics; these unadjusted and adjusted estimates should therefore be interpreted together rather than in isolation. Although PM_2.5_ and O_3_ were included in sensitivity analyses, other meteorological variables such as humidity and apparent temperature could confound associations. This qualitative discussion highlights that, despite controlling for major air pollutants, residual confounding from other environmental factors may remain and should be considered when interpreting the results. In addition, the animal model used continuous exposure to 33 ± 1 °C for 14 days, which represents sustained heat stress rather than the intermittent pattern of human heatwaves; the mechanistic findings in mice should therefore be regarded as a conceptual rather than a direct translation of population-level exposure. We were also unable to account for area-level or individual socioeconomic factors—such as neighborhood heat-island intensity, green-space coverage, housing conditions, or access to air conditioning—that may jointly shape personal heat exposure and health status, leaving open the possibility of residual socioeconomic confounding. Moreover, the survey data were collected in 2015, whereas the frequency and intensity of heatwaves have increased substantially since then; the present estimates may therefore not fully reflect current exposure–response relationships, and analyses incorporating more recent survey waves are warranted. The self-reported outcomes also encompass a heterogeneous range of gastrointestinal and hepatic conditions of differing severity and etiology (for example, from functional dyspepsia to advanced liver disease) that we were unable to disaggregate, and fatty liver was excluded because the questionnaire did not capture it; these factors limit mechanistic specificity and should be refined using diagnosis-coded or clinically validated outcomes in future work.

Nevertheless, our findings highlight the potential health risks of extreme heat exposure and motivate further mechanistic research into the effects of high-temperature environments on human health. They also support the development of heatwave early-warning systems and targeted protective measures for vulnerable populations, particularly smokers and middle-aged or older adults with pre-existing GI or liver conditions.

## 5 Conclusion

Using the CHARLS database, we explored the association between heatwave exposure and the prevalence of GI and liver diseases. Both the number of heatwave days and heatwave events were significantly associated with the prevalence of GI and liver diseases. Animal experiments showed that heat exposure induced gastrointestinal and hepatic injury, accompanied by alterations in the gut microbiome. These findings provide real-world and experimental evidence on the potential health burden of heatwave exposure and highlight the public health importance of developing targeted preventive and adaptive strategies.

## Data Availability

Data will be made available on request. 16S rDNA gene sequencing data were deposited in the BioProject database (PRJNA1344281).
