# Supplementary material for: Heatwave exposure and gastrointestinal and liver diseases: complementary evidence from a population-based study and animal experiments
Source: Front Physiol. 2026 Aug 6;17:1883010. doi: 10.3389/fphys.2026.1883010 (PMC13489915; doi:10.3389/fphys.2026.1883010)
Supplement: Supplementary file 1 [file SupplementaryFile1.docx]

Supplementary Material

## 1. Supplementary Figure

Supplementary figure 1: Sensitivity analyses adjusting for ambient air pollution (PM_2.5_ and O_3_). A: Heatwave days and GI diseases. B: Heatwave days and liver diseases. C: Heatwave events and GI diseases. D: Heatwave events and liver diseases.

Supplementary figure 2: Sensitivity analyses adjusting for ambient air pollution (PM_2.5_ and O_3_). A: Heatwave days and GI diseases. B: Heatwave days and liver diseases. C: Heatwave events and GI diseases. D: Heatwave events and liver diseases.

## 2. Supplementary Tables

Table S1. Descriptive analysis for twelve types of heatwaves definitions in the present study.

| **Heatwave definitions** | **Threshold percentile (℃) for temperature** | **Duration**  **(Day)** | **Intensity categories** |
| --- | --- | --- | --- |
| HW1 (90th-2D) | 90th | ≥2 | Low intensity |
| HW2 (90th-3D) | 90th | ≥3 | Low intensity |
| HW3 (90th-4D) | 90th | ≥4 | Low intensity |
| HW4 (92.5th-2D) | 92.5th | ≥2 | Middle-low intensity |
| HW5 (92.5th-3D) | 92.5th | ≥3 | Middle-low intensity |
| HW6 (92.5th-4D) | 92.5th | ≥4 | Middle-low intensity |
| HW7 (95th-2D) | 95th | ≥2 | Middle intensity |
| HW8 (95th-3D) | 95th | ≥3 | Middle intensity |
| HW9 (95th-4D) | 95th | ≥4 | Middle intensity |
| HW10 (97.5th-2D) | 97.5th | ≥2 | High intensity |
| HW11 (97.5th-3D) | 97.5th | ≥3 | High intensity |
| HW12 (97.5th-4D) | 97.5th | ≥4 | High intensity |

| **exposure** | **model1_OR** | | **model1_**  ***p*_value** | **model2_OR** | **model2_**  ***p*_value** | **model3_OR** | **model3_**  ***p*_value** |
| --- | --- | --- | --- | --- | --- | --- | --- |
| HW1_days | | 1.004  (1.001-1.007) | 0.003766 | 1.004  (1.002-1.007) | 0.002057 | 1.003  (1.00-1.006) | 0.02238 |
| HW2_days | | 1.006  (1.002- 1.01) | 0.004407 | 1.006  (1.002-1.011) | 0.00248 | 1.005  (1.001-1.009) | 0.02592 |
| HW3_days | | 1.008  (1.002-1.014) | 0.00507 | 1.008  (1.003-1.014) | 0.002905 | 1.006  (1.001-1.012) | 0.02909 |
| HW4_days | | 1.004  (1.001-1.007) | 0.004905 | 1.004  (1.002-1.007) | 0.002775 | 1.003  (1.00-1.006) | 0.02798 |
| HW5_days | | 1.006  (1.002- 1.01) | 0.00669 | 1.006  (1.002-1.011) | 0.003907 | 1.005  (1.0-1.009) | 0.03551 |
| HW6_days | | 1.008  (1.002-1.014) | 0.007233 | 1.008  (1.003-1.014) | 0.004254 | 1.006  (1.00-1.012) | 0.03756 |
| HW7_days | | 1.004  (1.001-1.007) | 0.006898 | 1.004  (1.001-1.007) | 0.004077 | 1.003  (1.00-1.006) | 0.03645 |
| HW8_days | | 1.006  (1.002- 1.01) | 0.009314 | 1.006  (1.002-1.011) | 0.00563 | 1.005  (1.00-1.009) | 0.04631 |
| HW9_days | | 1.008  (1.002-1.014) | 0.01007 | 1.008  (1.002-1.015) | 0.00618 | 1.006  (1.0-1.012) | 0.04898 |
| HW10_days | | 1.004  (1.001-1.007) | 0.01458 | 1.004  (1.001-1.007) | 0.00914 | 1.003  (0.9998-1.006) | 0.06999 |
| HW11_days | | 1.006  (1.001-1.011) | 0.0143 | 1.006  (1.002-1.012) | 0.009032 | 1.005  (0.9997- 1.01) | 0.07029 |
| HW12_days | | 1.008  (1.002-1.015) | 0.01707 | 1.008  (1.002-1.015) | 0.01104 | 1.006  (0.9994-1.013) | 0.08171 |

Table S2. Association between heatwave days and GI diseases.

Table S3. Association between heatwave days and liver diseases.

| **exposure** | **model1_OR** | | **model1_**  ***p*_value** | **model2_OR** | **model2_**  ***p*_value** | **model3_OR** | **model3_**  ***p*_value** |
| --- | --- | --- | --- | --- | --- | --- | --- |
| HW1_days | | 1.005  (1.002-1.008) | <0.001 | 1.005  (1.002-1.008) | <0.001 | 1.004  (1.001-1.007) | 0.006293 |
| HW2_days | | 1.008  (1.003-1.012) | <0.001 | 1.008  (1.004-1.013) | <0.001 | 1.006  (1.002-1.011) | 0.006936 |
| HW3_days | | 1.01  (1.004-1.016) | 0.001152 | 1.011  (1.005-1.017) | <0.001 | 1.008  (1.002-1.015) | 0.008143 |
| HW4_days | | 1.005  (1.002-1.008) | 0.001066 | 1.005  (1.002-1.009) | <0.001 | 1.004  (1.001-1.007) | 0.007518 |
| HW5_days | | 1.008  (1.003-1.012) | 0.001436 | 1.008  (1.004-1.013) | <0.001 | 1.006  (1.002-1.011) | 0.009497 |
| HW6_days | | 1.01  (1.004-1.017) | 0.00151 | 1.011  (1.005-1.018) | <0.001 | 1.008  (1.002-1.015) | 0.00978 |
| HW7_days | | 1.005  (1.002-1.008) | 0.001341 | 1.006  (1.002-1.009) | <0.001 | 1.004  (1.001-1.008) | 0.008929 |
| HW8_days | | 1.008  (1.003-1.013) | 0.001741 | 1.008  (1.004-1.013) | <0.001 | 1.006  (1.002-1.012) | 0.01103 |
| HW9_days | | 1.01  (1.004-1.017) | 0.001813 | 1.011  (1.005-1.018) | <0.001 | 1.009  (1.002-1.016) | 0.01128 |
| HW10_days | | 1.005  (1.002-1.009) | 0.002586 | 1.006  (1.002-1.009) | 0.001318 | 1.004  (1.001-1.008) | 0.01633 |
| HW11_days | | 1.008  (1.003-1.014) | 0.002499 | 1.009  (1.004-1.014) | 0.00129 | 1.007  (1.001-1.012) | 0.01625 |
| HW12_days | | 1.011  (1.004-1.018) | 0.002984 | 1.012  (1.005-1.019) | 0.001592 | 1.009  (1.002-1.016) | 0.01918 |

Table S4. Association between heatwave events and GI diseases.

| **exposure** | **model1_OR** | | | **model1_**  ***p*_value** | | **model2_OR** | **model2_**  ***p* _value** | **model3_OR** | **model3_**  ***p* _value** |
| --- | --- | --- | --- | --- | --- | --- | --- | --- | --- |
| HW1_events | | 1.058  (1.028- 1.09) | | <0.001 | 1.063  (1.033-1.095) | | <0.001 | 1.052  (1.021-1.083) | <0.001 |
| HW2_events | | 1.074  (1.035-1.115) | | <0.001 | 1.08  (1.04-1.122) | | <0.001 | 1.064  (1.024-1.106) | 0.0014 |
| HW3_events | | 1.081  (1.034-1.131) | | <0.001 | 1.086  (1.039-1.136) | | <0.001 | 1.068  (1.022-1.118) | 0.004115 |
| HW4_events | | 1.055  (1.027-1.085) | | <0.001 | 1.059  (1.03-1.089) | | <0.001 | 1.048  (1.019-1.078) | 0.001028 |
| HW5_events | | 1.066  (1.028-1.106) | | <0.001 | 1.071  (1.033-1.111) | | <0.001 | 1.056  (1.018-1.096) | 0.003784 |
| HW6_events | | 1.083  (1.034-1.135) | | <0.001 | 1.089  (1.04-1.141) | | <0.001 | 1.07  (1.022-1.122) | 0.004623 |
| HW7_events | | 1.057  (1.029-1.087) | | <0.001 | 1.061  (1.032-1.091) | | <0.001 | 1.05  (1.021- 1.08) | <0.001 |
| HW8_events | | 1.069  (1.029-1.113) | | <0.001 | 1.075  (1.034-1.119) | | <0.001 | 1.06  (1.019-1.103) | 0.004321 |
| HW9_events | | 1.087  (1.038- 1.14) | | <0.001 | 1.093  (1.044-1.147) | | <0.001 | 1.076  (1.026-1.129) | 0.002732 |
| HW10_events | | 1.051  (1.022-1.081) | | <0.001 | 1.055  (1.026-1.085) | | <0.001 | 1.045  (1.016-1.075) | 0.002249 |
| HW11_events | | 1.072  (1.032-1.115) | | <0.001 | 1.077  (1.037-1.121) | | <0.001 | 1.063  (1.023-1.106) | 0.002214 |
| HW12_events | | 1.072  (1.023-1.125) | 0.00385 | | 1.078  (1.029-1.132) | | 0.001868 | 1.062  (1.012-1.115) | 0.01479 |

Table S5. Association between heatwave events and liver diseases.

| **exposure** | **model1_OR** | | **model1_**  ***p*_value** | | **model2_OR** | **model2_**  ***p* _value** | **model3_OR** | **model3_**  ***p* _value** |
| --- | --- | --- | --- | --- | --- | --- | --- | --- |
| HW1_events | | 1.06  (1.029-1.093) | <0.001 | 1.067  (1.035-1.101) | | <0.001 | 1.054  (1.022-1.087) | <0.001 |
| HW2_events | | 1.08  (1.038-1.124) | <0.001 | 1.088  (1.046-1.133) | | <0.001 | 1.07  (1.028-1.115) | 0.001011 |
| HW3_events | | 1.09  (1.04-1.144) | <0.001 | 1.098  (1.047-1.152) | | <0.001 | 1.077  (1.027-1.131) | 0.002404 |
| HW4_events | | 1.058  (1.028- 1.09) | <0.001 | 1.063  (1.033-1.096) | | <0.001 | 1.051  (1.02-1.083) | 0.001088 |
| HW5_events | | 1.07  (1.03-1.112) | <0.001 | 1.077  (1.036- 1.12) | | <0.001 | 1.06  (1.019-1.103) | 0.003823 |
| HW6_events | | 1.089  (1.037-1.144) | <0.001 | 1.097  (1.045-1.153) | | <0.001 | 1.075  (1.024-1.131) | 0.004066 |
| HW7_events | | 1.06  (1.03-1.092) | <0.001 | 1.065  (1.035-1.098) | | <0.001 | 1.053  (1.022-1.085) | <0.001 |
| HW8_events | | 1.077  (1.033-1.123) | <0.001 | 1.084  (1.04-1.131) | | <0.001 | 1.067  (1.023-1.113) | 0.002833 |
| HW9_events | | 1.097  (1.044-1.154) | <0.001 | 1.106  (1.053-1.164) | | <0.001 | 1.086  (1.033-1.143) | 0.001516 |
| HW10_events | | 1.054  (1.024-1.086) | <0.001 | 1.06  (1.029-1.092) | | <0.001 | 1.048  (1.018- 1.08) | 0.001948 |
| HW11_events | | 1.079  (1.036-1.125) | <0.001 | 1.086  (1.043-1.133) | | <0.001 | 1.07  (1.027-1.117) | 0.001494 |
| HW12_events | | 1.083  (1.03- 1.14) | 0.002026 | 1.091  (1.038-1.149) | | <0.001 | 1.072  (1.019- 1.13) | 0.008166 |
